# Citizen Participation in Patient Prioritization Policy Decisions: An Empirical and Experimental Study on Patients' Characteristics

**DOI:** 10.1371/journal.pone.0036824

**Published:** 2012-05-09

**Authors:** Adele Diederich, Joffre Swait, Norman Wirsik

**Affiliations:** 1 Jacobs University, Bremen, Germany; 2 Centre for the Study of Choice, University of Technology, Sydney, Australia; 3 BIPS – Institute for Epidemiology and Prevention Research, Bremen, Germany; Johns Hopkins Bloomberg School of Public Health, United States of America

## Abstract

Health systems worldwide are grappling with the need to control costs to maintain system viability. With the combination of worsening economic conditions, an aging population and reductions in tax revenues, the pressures to make structural changes are expected to continue growing. Common cost control mechanisms, e.g. curtailment of patient access and treatment prioritization, are likely to be adversely viewed by citizens. It seems therefore wise to include them in the decision making processes that lead up to policy changes. In the context of a multilevel iterative mixed-method design a quantitative survey representative of the German population (N = 2031) was conducted to probe the acceptance of priority setting in medicine and to explore the practicability of direct public involvement. Here we focus on preferences for patients' characteristics (medical aspects, lifestyle and socio-economic status) as possible criteria for prioritizing medical services. A questionnaire with closed response options was fielded to gain insight into attitudes toward broad prioritization criteria of patient groups. Furthermore, a discrete choice experiment was used as a rigorous approach to investigate citizens' preferences toward specific criteria level in context of other criteria. Both the questionnaire and the discrete choice experiment were performed with the same sample. The citizens' own health and social situation are included as explanatory variables. Data were evaluated using corresponding analysis, contingency analysis, logistic regression and a multinomial exploded logit model. The results show that some medical criteria are highly accepted for prioritizing patients whereas socio-economic criteria are rejected.

## Introduction

The need for reform of healthcare provision in first-world countries is widely recognized, both in public and privately funded systems [1]. Aging populations, shifting demographics and social values, increasing costs and reductions in tax revenues are working together to deeply stress healthcare systems in Australia, Canada, France, Germany, Great Britain and the United States, among others. While specifics differ from country to country, controlling or reducing the cost of health care delivery takes a central role in the debate about reform in all of these places [2], [3].

With this background motivation, this paper addresses empirically the issue of prioritization of patient care, a necessary step to establishing service priorities, designing cost containment policies and imposing user fees. Most of the parties involved in healthcare reform debates – governments, politicians, healthcare professionals, pharmaceutical companies, special interest groups – actively work to make their desires known. Despite their obvious interest in this debate, however, it is the patients who will likely have the greatest difficulty in providing input to these discussions.

A common approach taken to policy formulation in the face of resource constraints is to adopt an utilitarian framework that seeks maximization of societal health benefits through reliance on the cost-effectiveness of health services (see discussion in [4]). However, as argued in [4], and supported by the health service prioritization exercise for the state of Oregon in the early 1990's, cost-effectiveness as a criterion does not seem to generate socially and politically palatable solutions due to the Rule of Rescue. This rule dictates that people cannot remain unresponsive or inactive when a specific, identified person's life is in peril and there exist effective means of “rescue” or aid. Hadorn [4] discusses in some detail one approach and experience to addressing this basic response in humans to the needs of other humans. We seek to introduce citizen participation into health prioritization dialogues as a complementary method that directly incorporates phenomena such as the Rule of Rescue and other psychological, emotional and social responses.

One controversial set of criteria for prioritizing health care concerns patients' personal characteristics. Whereas medical features such as severity of illness are generally supported as valid criteria for priority setting [5]–[7], it is less clear whether personal characteristics such as life-style and self-infliction of disease [8]–[10] or age [9], [11]–[13] are acceptable for the purpose. Research results have been inconsistent and seem to depend partly on the study design.

In this research we employ two different approaches used on the same representative sample of German citizens (N = 2031). First, we elicit the acceptance of possible prioritization criteria (medical, socioeconomic and lifestyle situation of the patient) via questionnaire items with binary responses to probe citizens' acceptance of broad and general criteria in a rather abstract way. Second, we investigate one rigorous mechanism, stated preference experiments [14] – also called discrete choice experiments (DCEs) – for injecting a “voice of the patient” (related to the long-standing “voice of the customer” concept in the marketing literature; see, e.g., [15]) into the healthcare reform debate. Here the criteria are more specific and a tradeoff between several criteria level is required to set a patient at the head of the priority list.

We illustrate the approach through a representative survey of German citizens, eliciting rankings of hypothetical patients (described by illness type and severity, demographics and lifestyle characteristics) in terms of service prioritization – who should be served first, second, etc. On the basis of this data, we relate reported patient service rankings to specified patient descriptions via a multinomial “exploded logit” [16] discrete choice model. This model is the mechanism whereby decision makers can “consult” the German population in terms of this specific issue when dealing with policy issues related to service prioritization. (See [17] for a similar focus with respect to public participation in transportation policy formulation.)

Discrete choice experiments have experienced increased acceptance in the health care research literature. We cite recent work to exemplify the penetration of DCEs in health research. Burge, Netten and Gallo [18], for example, use a DCE to estimate willingness-to-accept (WTA) valuations of a population for a large number of social care outcome domains. Johnson et al. [19] investigate the role of what they term a “recoding heuristic” to characterize how health care costs may be treated by subjects in DCEs due to distortions introduced by insurance coverage. Lancsar et al. [20] employ a DCE to derive distributional weights for quality adjusted life years (QALYs), based on characteristics of beneficiaries (see also [21]). To compare patient and physician perception of patient preferences concerning multiple myeloma therapy, Mühlbacher and Nübling [22] employ separate DCEs applied to the two respondent types; they find that there is broad agreement in preferences between the two groups. Green and Gerard [23] used DCEs for the process of health technology appraisal. As a final example, Scuffham et al. [24] proposed the use of DCEs as an aid to policy makers in designing health care system characteristics (e.g. health, equity, responsiveness and financing). Thus, the health care system literature has DCE applications with a variety of objectives: health system design, patient decision rules, comparative preferences between physicians and patients for a specific treatment, and support of economic valuations.

The DCE reported in this research is based on the same general approach employed in the above literature, but differs from them in that population tastes for preferential treatment decisions are elicited. In this sense, the purpose of this DCE is not to characterize the patient as the receiver of treatment in a health care system, but rather to permit the population's direct representation in the process of redesigning cost-related policies for their health care system.

In the remainder of the paper we discuss the survey structure and sampling methods that were employed, present an overview of the model estimation results, discuss the inferences that arise from the survey and model concerning German citizen preferences about service prioritization, then conclude with a discussion about the usefulness of methods such as stated preference experiments as mechanisms for bringing citizen preferences into public policy discussions.

## Materials and Methods

The reported methods and results are part of a more comprehensive study on prioritizing in medicine using a multilevel iterative mixed-method design (for details see, e.g., [25], [26]) for combining a qualitative interview study, a quantitative survey representative of the German public and focus groups. The quantitative survey included a DCE that will be described in due course. Approval for this study was granted by the Ethics Board of the University of Bayreuth (Ethik-Kommission für Forschungsfragen der Universität Bayreuth), 95440 Bayreuth, Germany.

### Sampling

The population survey was conducted in Germany by TNS Healthcare between July and September 2009, covering people aged 18 and over living in private households. Data were collected by computer assisted personal interviews (CAPI). The sampling followed a three-stage random route procedure, with a design developed by ADM (Association of German Market and Social Researchers). The first stage comprises electoral wards for national elections, the second the households, and the third the individuals within the target households selected by the Kish-table method (see, e.g., [27], [28], for details). Participants gave a verbal informed consent (i.e., agreed to participate) after they had been informed about the goals and content of the study, as well as about data protection and privacy. Participants' co-operation in this research project was entirely voluntary at all stages.

#### Material for the Questionnaire

A survey with 34 questions comprising 135 response items was organized around health care and health system related themes [28]. Both the topics addressed in the questionnaire and the attribute dimensions used for the discrete choice scenarios are based on results obtained from an exploratory interview study on prioritizing health care with 45 members of six different stakeholder groups [29]. One of the themes of the questionnaire was concerned with person-specific characteristics as possible criteria for preferential treatment.

The setting for this portion of the qualitative interview was established by the following statement: “*We would like to know whether a specific patient or specific patient groups should receive preferential treatment if medical services are not provided for by the public health insurance to the extent they used to be.”* If respondents asked for further clarification, the interviewer stated that preferential treatment meant that this specific person would be treated first, but did not mean that other patients were not to be treated at all. That is, patients might receive treatment later or with fewer resources.

A first block of questions described patients in an abstract fashion with only one person characteristic. Responses to the question “Do you think it is justifiable to treat the following patient groups in preference to all others?” were provided for 18 different groups: 1) people who are active in the community (e.g. volunteer workers); 2) patients with a life-threatening disease/illness; 3) senior citizens; 4) patients with psychological illness; 5) people with a healthy lifestyle; 6) people with high income; 7) patients with chronic illness; 8) children; 9) patients with a low quality of life; 10) people with high level of professional responsibility (e.g. people in an executive position supervising several employees); 11) patients with physical handicaps; 12) patients with acute diseases; 13) socially disadvantaged people; 14) people with children; 15) people of working age; 16) patients with mental handicaps; 17) unemployed people; 18) people with social responsibilities (e.g. caring of relatives). The response categories included a “Yes” and “No” response; “Don't know” and “Response refused” options were offered only when the person did not respond with one of the first two categories.

In addition to questionnaire items, two different discrete choice experiments [14] were presented. One experiment described patients with several person characteristics (specified below), whereas the second dealt with other new treatments not considered in this paper. The questionnaire ended with socio-demographic questions and a self-report on the respondent's life-style and on health, the latter measured by the Short-Form Health Survey (SF-8™) [30].

#### Material for the Discrete Choice Experiment

The discrete choice experiment requires construction of choice alternatives (called “profiles”) which are characterized by several attributes and different levels (or values) on these attributes. In the current context the choice alternatives are hypothetical patients.

The following attributes and their levels were used to create the profiles (i.e. person-condition descriptions) for the discrete choice experiment (DCE).

#### Health Status (levels: severe disease, light disease)

This attribute summarizes a large number of diseases and injuries. Purposely no concrete examples were given to avoid personal conflict. We relied on a general agreement among all participants that all (acute) life threatening diseases, emergencies and diseases that generally lead to death (e.g. cancer or heart failure) are severe diseases, whereas non-life threatening and most chronic diseases are considered light illnesses.

#### Quality of life (levels: severely restricted, restricted, no restrictions)

The categories followed the EuroQol classification [31]: a strongly restricted person is unable to perform usual activities and has extreme pain and discomfort; a restricted person has some problems in usual activities and moderate pain and discomfort; and a person with no restrictions is in full health with no restrictions in usual activities.

#### Unhealthy life style (levels: yes, no)

The attribute is intended to address the person's individual responsibility to care for his/her health. An unhealthy lifestyle includes smoking, unhealthy diet, excessive drinking, no physical exercise or excessive sun bathing.

#### Age of patient (levels: 25, 43, 68, 87 years)

Each level is representative of an age group and reflects the age structure of adults (over 18 years) in Germany. The level 25 years represents the group of young adults in their early career; the level 43 years represents the middle aged adults, mainly with a settled career; the level 68 years represents retired people and the level 87 years the old-age group.

#### Family status (levels: single with/out dependents (children, relatives to care for), couple with/out dependents (children, relatives to care for))

For plausibility the description with/out dependents was specified as single/couple with/out children when the age in the profile was 25 or 43 years and single/couple with/out family members to care for when the age in the profile was 68 or 87 years. Couple means living together with a partner and includes all kinds of partnerships.

#### Occupational status (levels: high, medium, low)

The attribute served as a proxy for socioeconomic status. A high occupational status includes CEOs, physicians, lawyers; a medium status includes clerical workers and craftsmen; low status includes unskilled workers and long-term unemployed people. For profiles with levels 68 or 87 years, the occupational status refers to the time before retirement.

### Experimental Design: Generation and presentation of patient profiles

With six attributes, two of them having two levels, two with three levels, and two with four different levels, 576 ( = 4^2^×3^2^×2^2^) different profiles are possible. The number of profiles was reduced to 23 by applying an orthogonal fractional factorial main effects design, utilizing the SAS PROC OPTEX routine which ensures d-optimality. Combining three profiles into one choice set implies there are 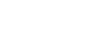 possible sets; a subset of 25 choice sets was selected using the SAS %choiceff macro which also ensures d-optimality (see [32] for procedural details). Of these 25 choice sets, each respondent saw four different randomly selected choice sets which were embedded in the study's questionnaire. All profiles were presented as full profiles (i.e. all attributes presented); the participant had to indicate which hypothetical patient should be treated first and which patient should be treated last. Table 1 illustrates how the choice set was presented to respondents, including the settings preface.

**Table 1:** Illustrative Choice Set in Discrete Choice Experiment.

Patient Characteristic	Patient A	Patient B	Patient C
Occupational Status	high	medium	low
Health status	light disease	severe disease	severe disease
Quality of life	severely restricted	restricted	no restrictions
Unhealthy life style	yes	yes	no
Age	25	43	87
Family status	single with child	single with child	single with no relatives to care for
	**Which of the patients would you treat first?**		
	□	□	□
	**Which of the patients would you treat last?**		
	□	□	□

*Above we introduce three patients with different characteristics. Which of the patients would you prefer be treated first and which last?*

Limiting the choice sets to four within one questionnaire was done to minimize cognitive burden. Obviously that limits the amount of data provided by any single respondent: instead of 25·N total choice responses only 4·N are obtained, where N is the number of participants in the survey. However, since the choice task produces the ranking of the three hypothetical patients described, the information content in the preference rank ordered choice sets can be exploited to double the amount of data [16].

## Analysis

### Socioeconomic status

The socioeconomic status was determined by the “Winkler-Index” [33]. This measure is a three-dimensional, additive, non-weighted social class index using academic/vocational education, monthly net household income and current/last occupation as indicators. Each indicator ranges from one to seven points, where one point represents the lowest and seven the highest social status; hence the Winkler-Index can take values between three and 21 points. Three social status groups with equal ranges can be defined on the basis of this index: lower status (3–8 points), middle status (9–14 points) and higher status (15–21 points) [34].

### Health status

The SF-8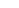 Health Survey produces a physical (PCS) and a mental (MCS) component summary measure. Based on the scores and according to the instrument norm of 50 [35] each participant was categorized as average and above (score≥50) and below average (score<50), separately for each component. In addition, the sample medians were taken to categorize the participants in above and below average.

### Family status

The family status of the sample was inferred from the combination of three questions in the survey: participants indicated their 1) marital status, 2) partner status (yes/no), and 3) number and age of persons living regularly in the household.

### Lifestyle

The lifestyle measure we employ is based on 1) smoking habits (non-smokers vs. smokers); 2) alcohol consumption habits (none/little, moderate, heavy); 3) weight and height of participants, converted to the Body Mass Index (BMI) (underweight (BMI<18.5), normal weight (18.5≤BMI<25), overweight (25≤BMI<30), obese (BMI≥30); 4) body exercise habits (often - three and more times a week, moderate - one to two times per week, and never/seldom). Each category value was assigned one, two and three points, where one point represents behavior that presumably does not affect health negatively, and three points represents behavior that presumably affects health most negatively. A lifestyle index was determined by adding the points. The lifestyle category was defined on the basis of this index: healthy (4–5 points), average (6–7 points), and unhealthy (8–12 points). Note that the categories for non-smoking and smoking received one and three points, respectively.

### Survey data

A multiple correspondence analysis [36] was performed on the overall results to discover patterns of criteria of acceptance. To investigate the influence of respondents' age, sex, socio-economic status, health status, family status, and lifestyle on attitudes towards patient characteristics, we conducted a supporting contingency analysis with adjusted residuals as follow-up chi-square tests. An adjusted residual that exceeds about 2 or 3 in absolute value indicates a lack of fit of H_0_ in that cell [37]. Here we applied them as posthoc tests with a 5% (adjusted residual = 2) and 1% (adjusted residual = 3) significance level. A binary logistic regression analysis was carried out with age, PCS (physical health status), and MCS (mental health status) as covariates, and socio-economic status and lifestyle as factors.

### Utilities, choice probabilities, and relative importance

Data for the discrete choice experiment were analyzed using a random utility model for rank ordered data [16]. The utility 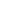 of the k^th^ profile in the l^th^ choice set is determined by the sum of the partworth utilities 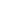 of attribute i with level j, perturbed by error 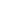, i.e.,
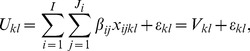
(1)where 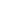 indicates whether profile 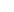 has attribute i with level j (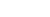) or not (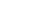). The error components are identically and independently distributed Gumbel (double exponential) variates with zero mean and variance π^2^/6. The probability that choice alternative (profile) k of the l^th^ choice set is chosen is therefore given by
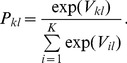
(2)


According to the rank order explosion rule [16], the rank ordered observations can be exploded (decomposed) into statistically independent choice observations under these assumptions. For instance, adding the observed frequency of the rankings of the patients 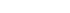 and 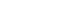 (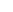 means “is preferred to”) gives us the frequency ranking A as first out of the choice set {A,B,C}. The observed frequency of the ranking 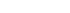, 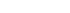 and 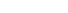 gives the frequency for ranking A first out of the choice set {A,B}.

The relative importance of attribute i is defined in terms of the range of its partworth utility values relative to the partworth utility ranges of all attributes:
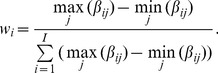
(3)


Data of all participants were aggregated to obtain this measure.

The random assignment of four choice subsets out of the 25 possible choice sets to a questionnaire does not necessarily lead to a balanced distribution of choice sets over completed questionnaires. This may lead to heterogeneous precision for the partworth estimates of different attribute levels. To assess this possibility and adjust the estimates we simulated a null model by assigning random answers to the observed (distribution of) choice sets. The procedure was performed on the entire data set and on population subgroups. The confidence intervals (CI) for the attribute levels are directly obtained from the covariance matrix; the 95% (CI) for the relative importance were determined via bootstrap sampling: 1000 random samples with 2031 observations were drawn from the original data set (N = 2031). For each random sample we calculate the relative importance of the attributes. The 1000 estimates of relative importance were ordered by size and the 25th and 97.5th estimates were used as estimators for the 2.5% and 97.5% quantiles, respectively, and hence the lower and upper limit of a 95% confidence interval.

## Results

### Sample Description

The number of selected addresses was 3729, of which 3% were ineligible (e.g., no private household). Of the remaining 3617 addresses, 22% of the target persons were unavailable, 13% refused to take part and 8.2% were unable to do so for other reasons (e.g., illness), resulting in a response rate of 56.8% (2031 respondents). The sample is representative for the adult population (18 years and above) of Germany. It includes 1131 (55.6%) female and 900 male respondents. Mean, median and standard deviation of their age is 52, 52, and 18 years, respectively. For the analysis, respondents are grouped into three age groups: 18–29 years (14.1% of the sample), 30–59 years (46.7% of the sample) and 60 years and above (39.2% of the sample). The first group represents young adults in their early career; the second the working age group and the last the elderly. The average de facto retirement age is 60 years in Germany.

#### Socio-economic status

According to the Winkler-index, 47% of the respondents belong to a lower social status, 39.8% to a middle social status, and 13% to a higher social status groups. Three participants (0.2%) did not give any information and therefore could not be classified.

#### Health status

According to the SF-8™, 64.8% of the respondents have a physical health score of PCS≥50, i.e., average and above, and 78.2% a mental health score of MCS≥50. The sample median for the PCS and the MCS was 54.2 and 57.3, respectively, slightly higher than for the reference group (General U.S. population, in 2000, with 51.9 and 51.1, respectively), and therefore, we decided to use the sample median for the subsequent analyses.

#### Family status

The distribution of family status according to the categories described above is as follows: 30.1% of the sample are single; 7.1% are single with child (children) living in the same household; 41.9% of the sample live in a partnership without children in the same household and 20.9% in a partnership with child (children) in the same household.

#### Lifestyle

The lifestyle distribution measured in term of smoking habits, alcohol consumption, BMI, and exercise, is as follows: 29.8% of the sample are smokers and 71.2% non-smokers; 48.9% of the sample reported none/little, 49.2% moderate and 1.9% heavy alcohol consumption. According to the BMI statistic, 1.5% are underweight, 46.9% have normal weight, 37.9% are overweight, and 13.7% are obese. The underweight group was merged with the normal weight group. In terms of exercise habits, 31.3% of the sample never/seldom exercise, 28.9% exercise moderately, and 39.8% exercise often. According to the lifestyle index, 23.0% of the sample live healthy lifestyles, 48.5% are of average health lifestyle, and 28.5% have an unhealthy lifestyle. Table 2 reports the sample statistics.

**Table 2:** Summary of the sample statistics based on N = 2031 participants.

Characteristics	n	%
**Sex**		
Female	1131	55.6
Male	900	44.4
**Age**		
18–29	287	14.1
30–59	948	46.7
≥60	796	39.2
**Socio-economic status**		
Lower	955	47.0
Middle	808	39.8
Higher	265	13.0
**Health status**		
PCS≥50 (Test norm)	1316	64.7
PCS<50	715	35.3
PCS≥54.2 (Sample median)	931	45.8
PCS<54.2	1100	54.2
MCS≥50 (Test norm)	1588	78.2
MCS<50	443	21.8
MCS≥57.3 (Sample median)	1014	49.9
MCS<57.3	1017	51.1
**Family Status**		
Single	611	30.1
Single with children	144	7.1
Partnership	851	41.9
Partnership with children	425	20.9
**Lifestyle**		
Healthy	467	23.0
Average	985	48.5
Unhealthy	579	28.5

### Aggregate Characterization of Preferential Treatment Results

The majority of respondents agreed to preferential treatment for patients with a life-threatening disease, patients with acute diseases, children and patients with physical handicaps. Features that reflect both the socio-economic status (i.e., income, unemployment, professional obligations) and social engagement outside the family are clearly rejected as criteria for preferential treatment (Table 3).

**Table 3:** Proportion of agreement/disagreement to the question “Do you think it is justifiable to treat the following patient groups in preference to all others?” with 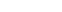 respondents.

	Response categories			
Criterion	Yes	No	Don't know	Answer refused
Life-threatening disease	93.7	5.8	0.4	0.0
Acute diseases	87.2	11.3	1.4	0.1
Children	72.5	25.4	1.9	0.2
Physical handicap	57.0	38.7	3.9	0.3
Senior citizens	50.2	45.4	4.0	0.3
Low quality of life	49.1	45.1	5.5	0.3
With children	46.4	49.4	3.7	0.4
Mental handicap	43.9	51.0	4.9	0.2
Psychological illness	42.5	51.4	5.7	0.4
Chronic illness	42.3	54.2	3.4	0.1
Social responsibility	31.4	65.1	3.3	0.1
Working age	14.4	83.7	1.9	0.1
Socially disadvantaged	13.8	83.2	2.9	0.2
Healthy lifestyle	8.4	88.9	2.5	0.2
Active in the community (socially active)	5.9	92.4	1.7	0.0
Professional responsibility	5.8	92.9	1.2	0.1
Unemployed	4.8	93.7	1.4	0.1
High income	1.6	97.7	0.5	0.1

**Figure 1:**
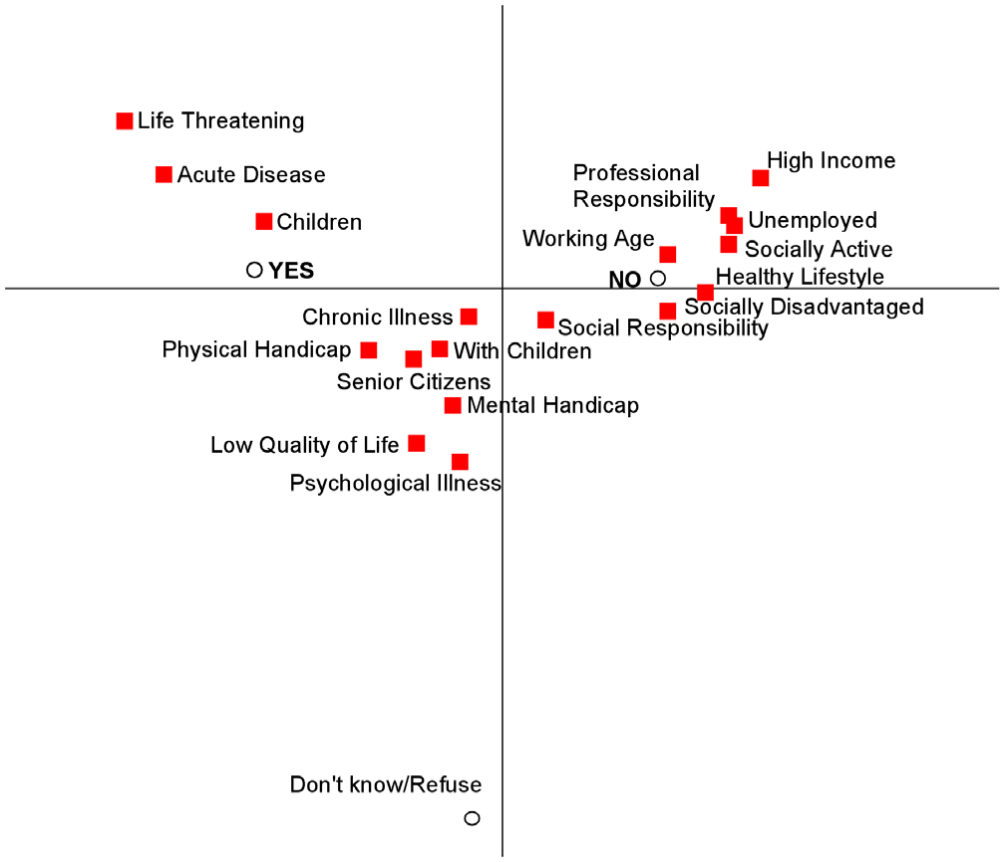
Biplot of Preferential Treatment Survey Results.

A multiple correspondence analysis identified three distinct groups on an acceptance - nonacceptance dimension for prioritization criteria (Figure 1). The response categories “Don't know” and “Answer refused” are merged due to the low frequencies in each category. The biplot shows that the more extreme the proportion of overall agreement/disagreement, the smaller is the proportion of uninformative responses. The criteria in the middle cluster of the biplot, which includes health issues as well as some social issues, seems more controversial to respondents (alternatively, more heterogeneous across respondents). Interestingly, patients with psychological illness and mental handicaps received less support for preferential treatment than patients with physical handicaps.

The contingency analysis revealed that some respondents' characteristics influenced the preferences for some prioritization criteria and some other characteristics such as gender and family status had no influence on preferences (Table S1 for details). The adjusted residuals show statistically significant differences in the response patterns as functions of the respondents' specific characteristics (Table S2 for details).

- Overall, healthy participants (health status median and above) disagree more often with preferential treatment for patients with mental handicaps, of working age, active in the community or with professional or social responsibility than do participants with a below median health status. Furthermore, participants with a median and above MCS score disagree more often with preferential treatment for patients with low quality of life, physical or mental handicap than do participants with a below median MCS score. On the other hand, healthy participants agree more often on preferential treatment for patients with a life-threatening disease than participants with a below average health.
- Respondents in the middle age group (30–59 years) disagree more often with preferential treatment for patients with psychological illness and patients who are active in the community than the younger and older respondents. Younger participants (18–29 years) agree more often on preferential treatment for patients with mental handicaps.
- Respondents with a lower socio-economic status tend to agree more often with preferential treatment for senior citizens and people of working age and for patients with mental handicaps, psychological illnesses and chronic illness than participants with a middle and high socio-economic background; participants with a higher socio-economic status disagree more often with preferential treatment for senior citizens and patients with mental handicaps, psychological illnesses and chronic illness than low and middle status participants. Participants with a lower socio-economic status disagree more often with preferential treatment for persons with professional responsibilities whereas participants with a middle socio-economic status more often agree with preferential treatment of this group.
- Persons with a healthy lifestyle tend to agree more often with preferential treatment and people with an unhealthy life style disagree more often with preferential treatment for patients with mental handicaps.

The results of the logistic regression showed significant main effects of age, socio-economic health status, both physical and psychological, and lifestyle for some of the criteria.

- The older the participants are the less they agree to preferential treatment of children, people with children and patients with mental handicaps.
- The higher the participants score on the MCS (mental health) the less they agree with preferential treatments of patients with a low quality of life, patients with mental handicaps, patients with social or professional responsibilities, socially disadvantaged or unemployed patients. On the other hand, the higher the score the more often they agree to preferential treatment of patients with life threatening diseases.
- The higher the participants score on the PCS (physical health) the less often they agree to preferential treatment of patients with mental handicaps.
- The lower the socio-economic class of participants, the more often they agree to preferential treatment of children, senior citizens, and people with children and patients with psychological diseases or with mental handicaps.
- The healthier the lifestyle of participants, the more often they agree to preferential treatment of children, senior citizens, people with children and people with social responsibilities, patients with psychological diseases, patients with mental handicaps and patients with a healthy lifestyle (Tables S3 and S4 for details).

Very few binary interactions were observed, mainly between socio-economic status and lifestyle, health status and lifestyle and between physical and psychological health status.

### Partworth utilities and relative importance

Of the 2031 participants, 1915 completed all four presented choice sets and 38 none. The remaining 78 participants did respond to all but one choice pair. This high completion rate (94.3% of respondents completed all choice sets; another 3.8% completed 3 of 4 choice sets) is indicative of respondents' high level of involvement with the survey and topic, as well as their willingness to reveal personal preferences on potentially sensitive topics. The analysis is based on 15866 individual choices, arising from the ranking of three patient profiles in each choice set. Table 4 summarizes the partworth utilities for each level (i.e. value) of the attributes, and the consequent relative importance for each attribute; confidence intervals for these measures are provided. Attributes are ordered according to aggregate relative importance.

**Table 4:** Partworth Utilities For Each Attribute Level And Relative Importance Of Attributes.

Attribute	Utility*	95% CI	Importance	95% CI
**Health status**			50.0%	(47.7% , 52.0%)
light disease	−0.483	(−0.500 , −0.465)		
severe disease	0.483	(0.466 , 0.500)		
**Quality of life**			24.7%	(22.9% , 26.3%)
no restrictions	−0.262	(−0.286 , −0.238)		
restricted	0.047	(0.023 , 0.072)		
severely restricted	0.215	(0.193 , 0.236)		
**Age**			12.0%	(10.1% , 14.0%)
25 years	0.052	(0.021 , 0.082)		
43 years	0.086	(0.058 , 0.113)		
68 years	0.009	(−0.018 , 0.036)		
87 years	−0.147	(−0.176 , −0.118)		
**Family status**			7.9%	(6.0% , 9.8%)
single w/o dependents	0.0081	(−0.019 , 0.035)		
single with dependents	0.086	(0.057 , 0.115)		
couple w/o dependents	−0.067	(−0.095 , −0.039)		
couple with dependents	−0.027	(−0.056 , 0.002)		
**Occupational status**			4.6%	(2.9% , 6.2%)
high	−0.038	(−0.062 , −0.014)		
medium	−0.013	(−0.037 , 0.011)		
low	0.051	(0.029 , 0.073)		
**Unhealthy life style**			0.8%	(0.04% , 2.3%)
yes	0.008	(−0.008 , 0.024)		
no	−0.008	(−0.024 , 0.008)		

* Estimation by maximum likelihood method, SAS PROC PHREG, option ties = breslow ([32]).

*Health status* is by far the most important attribute (relative importance: 50.0%). Not surprisingly a severe disease makes it more likely that a patient should be treated before a patient with a light disease, holding all other attributes constant. *Quality of life* is the second most important attribute but gets only half of the importance score for health status (relative importance: 24.7%). The more restricted a patient is in his or her every day activities, the higher the agreement for preferential treatment of this patient. The relative importance for *age* is 12.0%. The weight is about half that of the previous attribute and about a quarter of the most important attribute, health status. The most preferred age was 43, which represents people of working age. The partworth utilities decrease for both decreasing and increasing age, with a steeper decrease for increasing age. Taken together, the remaining three attributes account for about the same importance as age by itself. *Family and occupational status* represent the socioeconomic background of the hypothetical patients, as well as level of social responsibility. With relative importance values of 7.9% and 4.6% respectively, these attributes play only a minor role in determining preferential treatment. Patients with social responsibilities, i.e., caring for dependents, are preferred to those without caring obligations. Within this group, singles are preferred to couples. Couples without dependents receive the lowest partworths. Even less important for determining priority treatment is the patient's economic status, i.e. his or her occupation: the patient with the lowest status is preferred over the one with the highest status. The relative importance weight of attribute *Lifestyle* is negligible (0.8%) and the partworth utilities are not significantly different from zero.

From the above we can derive a rank order in which patients should be treated, according to the estimated model. For instance, a 43 year old patient with a severe disease and a severely restricted quality of life who has an unhealthy life style, comes from a low socio-economic background (occupation) and is a single parent gets the highest agreement in being treated first. We will refer to this hypothetical patient as the *reference patient*. The lowest rank is attributed to an 87 year old patient with a light illness and no restrictions in his/her quality of life, has a healthy life style, lives together with a partner without having social responsibilities for others and comes from a high socio-economic class. The first-treatment probabilities (Eq. 2) for choosing between these two archetypal patients are 0.87 for the reference patient and 0.13 for the other patient. To see the impact of each attribute level it is possible to vary one attribute while keeping the others constant (Table 5). For instance, the probability that the reference patient should be treated first versus that of another patient who has the same characteristics except for the health status (light) is 0.72 for the reference and 0.28 for the other patient. The difference in probabilities is quite substantial and reflects the importance of the attribute health status for preferential treatment ranking. However, varying the patient's age and keeping the remaining attribute levels fixed as before yields very small changes: the probability to be treated first is 0.26 for the 25 years old patient, 0.27 for the 43 year old patient (the reference patient), 0.25 for the 68 year old patient and 0.22 for the 87 year old patient. Although age contributes 12% to the overall importance, it is clear that its impact on ranking probabilities is minor. Note that we obtain basically the same result when the patient with the lowest rank is taken as the reference patient.

**Table 5:** Estimated Preferential Treatment Probabilities With Respect to Reference Patient.

Attribute	Probability
**Health status**	
light disease	0.28
severe disease	0.72
**Quality of life**	
no restrictions	0.25
restricted	0.34
severely restricted	0.41
**Age**	
25 years	0.26
43 years	0.27
68 years	0.25
87 years	0.22
**Family status**	
single w/o dependents	0.25
single with dependents	0.27
couple w/o dependents	0.24
couple with dependents	0.24
**Occupational status**	
high	0.32
medium	0.33
low	0.35
**Unhealthy life style**	
yes	0.50
no	0.50

We estimated the parameters for the rank-ordered logit separately for each of the aforementioned respondents' characteristics (age, socioeconomic status, health status (PCS), family status, and lifestyle). No statistically significant differences could be observed between the parameters of the respective groups for any of the attributes.

## Discussion

The steadily growing demand for health care provision on one hand and limited financial resources of the healthcare system on the other hand - whether publicly or privately financed - is a challenge for many countries of the OECD and beyond [1]. Priority setting in health care services is being discussed as a possibility to overcome the problem and to provide a fair distribution of resources in many countries ([3] for a recent review). Indeed, it is already being practiced in several countries like Sweden or England. In Germany, a broad and public discussion on this topic has yet to occur. Health insurance is mandatory in Germany for all citizens and nearly 90% of the population is covered by the Statutory Health Insurance (the remaining citizens are otherwise insured). The amount of insurance contributions mainly depends on the gross income of the insured person and is co-financed by employer and employee. The claim for benefits is independent of the amount of insurance contributions. Children and non-earning spouses are exempt from paying a premium and are covered by the so called family coinsurance. (For details on what is covered and how the system is financed see [38]). Measured in terms of GDP, health expenditure in Germany, all in all, is fourth after the US, Switzerland and France.

To probe the acceptance of priority setting in medical treatment decisions, a quantitative survey representative of the German public (N = 2031) was conducted. The present study focused on person characteristics as possible criteria for setting priorities. We investigated several criteria - both related and unrelated to a person's health (e.g., severity of disease, responsibilities for dependents) - with questionnaire items and discrete choice experiments. Unlike other studies (see [39] for a literature review), all attributes describing the hypothetical patients for the DCE were purely person-related (occupational status, health status, quality of life, unhealthy life style, age, family status) and not presented together with attributes describing treatments such as cost, therapeutic benefit, cost-effectiveness relations, and disease-related aspects, such as disease frequency or specific diseases. Furthermore, both the questionnaire and the DCE were performed within the same sample. To the best of our knowledge, this is the first study to conduct face-to-face interviews, including a DCE and supporting survey questions, with a representative sample to obtain preferences in a medical treatment priority-setting context.

Several explanatory variables were included to account for potential differences in preferences for patient prioritization: the interviewee's age, sex, socioeconomic status, health status, and lifestyle.

The results of the survey questions showed that the vast majority of respondents agreed to prioritize patients with life threatening diseases and patients with acute diseases over all other patients. All criteria that described the patient's social engagement outside the family or socio-economic status (e.g., income, unemployment) were rejected as possible criteria for prioritization. A similar pattern could be observed in the discrete choice experiment: health status received the highest importance weight, whereas socio-economic status received a very low weight in terms of deciding which patient should be treated first. There is considerable agreement that those in need, i.e., the severely ill patient, should be treated first [1], [5]–[7]. Socio-economic status was not considered acceptable, but is a commonly practiced criterion in the daily routine of physicians [40], if not explicitly, at least implicitly so [41], [42].

Lifestyle or self-infliction of disease have become prominent criteria when discussing priority setting in health care resources, since individual responsibility seems a reasonable and plausible criterion for health care allocation [8], [9], [43]. On the other hand, it is difficult to determine whether specific health conditions are caused by an unhealthy lifestyle rather than by genetic, societal or environmental factors [43]. Furthermore, socioeconomic factors may a) influence the adoption of a specific lifestyle [44] and b) lead to posteriorizing patients with an unhealthy lifestyle; this may contribute to more social class-health inequalities [45]. (Posteriorizing is the opposite of prioritizing, i.e., limiting access to medical services.) In the present DCE the lowest weight was given to lifestyle, in agreement with the results of the questionnaire. Note that in the former case the attribute was described as a lack of a healthy lifestyle (unhealthy) whereas in the latter it was positively phrased (healthy). That is, in this study we find that lifestyle neither serves as punishment nor as reward when assigning health treatment priorities. Interestingly, however, in the same questionnaire the majority of respondents supported copayments for medical services for patients with harmful behavior such as drug consumption, e.g., heroin (76.4%); extreme sports, e.g., free climbing, cliff diving (74.7%); high alcohol consumption (70.9%); smoking (67.8%); sunbathing/solarium (65.0%) (see [10] for details). These apparent discrepancies may be interpreted as follows. For the preferential-treatment-of-persons question as well as for the DCE, lifestyle was described abstractly as healthy and unhealthy. In contrast, the description of the health behavior in the copayment question was concrete and even illustrated by examples. The respondents obviously evaluated the described behavior differently and the result may well be an effect of framing as observed in other studies [46], [47]. Furthermore, the preferential-treatment-of-persons question and the DCE aimed at a preferential treatment, i.e., some patients are treated and others are not (yet). The situation is different for the copayment question: all patients are treated, but patients with some specific unhealthy behavior need to contribute out of pocket to the medical service [10].

Age, a highly disputed criterion for prioritizing medical services, is also controversial in this study. The majority favored children (72.5%) and elderly (50.2%) to be preferentially treated compared to all other patients; only a few respondents (14.4%) opted for preferential treatment for patients of working age. A detailed analysis, taking into account seemingly inconsistent response behavior, i.e., respondents agreed to preferential treatment to all others for two or all three age groups simultaneously, revealed that only a few respondents had a “true” preference for treating patients of a specific age group prior to all others (in particular, 24.7% favored only children, 6.5% only elderly and 0.7% only persons of working age - see [11] for details and an extensive literature discussion on age as criterion for prioritizing health care). This result is also reflected in the DCE data. The relative importance of age is 12%; its impact on ranking patients, however, is negligible.

In the survey data, differences in preferences for specific groups were partly explained by the respondents' own characteristics, for instance, socio-economic and health status. Those with a low status tended to agree more often to preferential treatment of some specific patient groups than of participants with a high socio-economic status. Healthy participants tended to agree less with preferential treatments than participants with a low level of health. This could not be observed for the DCE: the importance weights for prioritization criteria did not depend on the respondents' own characteristics. A major advantage of the DCE over questionnaire items is that respondents consider several attributes jointly, compare them, and make trade-offs to reach a decision. Apparently, the influence of self-interest is less pronounced when criteria are considered in context rather than in isolation. The potential for strategic behavior by respondents is often cited by critics of DCE's as a fatal shortcoming; however, simultaneous consideration of multiple attributes militates against such behavior and aids the revelation of true preferences (see, e.g., chapter 13 of [14]).

Taken together, the results show that there is substantial consensus among the German citizenry concerning what can and cannot serve as prioritization criteria for health services. In particular, medical criteria are highly accepted for prioritizing patients whereas socio-economic criteria and lifestyles are rejected. Especially the DCE showed that health status and quality of life were the only attributes that respondents would ultimately likely include in a decision-making process about which patients to prioritize for care. Policy makers in Germany have been very reluctant to even discuss the topic; indeed, all ministers of health over the last decade or so have refused to even talk about this issue. The present study shows that the “voice of the patient” – reliably captured through the methods used here – can be encapsulated in statistical models and thus introduced into policy-making settings [15]. The methods and findings illustrated in this research can be used to 1) increase citizen participation in the political discussion concerning this substantive policy topic, 2) define the scope of policy actions within the realm of the feasible, and 3) frame communications between policy-setting bodies and the population.

## Supporting Information

Table S1 Contingency analysis.
(DOC)

Table S2 Adjusted residuals.
(DOC)

Table S3 Logistic regression.
(DOC)

Table S4 Odds ratio.
(DOC)
