# Supplementary material for: Citizen Participation in Patient Prioritization Policy Decisions: An Empirical and Experimental Study on Patients' Characteristics
Source: PLoS One. 2012 May 9;7(5):e36824. doi: 10.1371/journal.pone.0036824 (PMC3348901; doi:10.1371/journal.pone.0036824)
Supplement: Table S1 — Contingency analysis. (DOC) [file pone.0036824.s001.doc]

Table S1: Significant results of the contingency analysis (Chi Square values, p-values).

|  | Respondents’ characteristics | | | | |
| --- | --- | --- | --- | --- | --- |
| Criterion | Age | Socio-econ | Health Status | | Life Style |
|  |  |  | PCS | MCS |  |
|  | df = 4 | df = 4 | df = 2 | df = 2 | df = 2 |
| Life-threatening disease |  | 14.7, 0.005 |  | 9.8, 0.007 |  |
| Physical handicap |  |  |  | 9.9, 0.007 |  |
| Senior citizens |  | 21.2, 0.000 |  |  |  |
| Low quality of life |  |  |  | 8.1, 0.017 |  |
| Mental handicap | 13.4, 0.010 | 10.5, 0.033 | 10.0, 0.007 | 14.5, 0.001 | 10.3, 0.036 |
| Psychological illness | 10.9, 0.028 | 17.9, 0.001 |  |  |  |
| Chronic disease |  | 9.5, 0.050 |  |  |  |
| Social responsibility |  |  | 10.9, 0.004 | 13.1, 0.001 |  |
| Working age |  | 10.5, 0.033 | 8.2, 0.016 | 10.4, 0.006 |  |
| Socially disadvantaged |  |  |  | 8.1, 0.018 |  |
| Active in the community | 9.9, 0.042 |  | 6.6, 0.037 | 6.8, 0.034 |  |
| Professional responsibility |  | 12.7, 0.013 | 10.7, 0.005 | 16.3, 0.000 |  |
| Unemployed |  |  |  | 9.3, 0.009 |  |
