# Supplementary material for: Citizen Participation in Patient Prioritization Policy Decisions: An Empirical and Experimental Study on Patients' Characteristics
Source: PLoS One. 2012 May 9;7(5):e36824. doi: 10.1371/journal.pone.0036824 (PMC3348901; doi:10.1371/journal.pone.0036824)
Supplement: Table S2 — Adjusted residuals. (DOC) [file pone.0036824.s002.doc]

Table S3: Adjusted residuals utilized as posthoc tests with a 5% (adjusted residual = 2) and 1% (adjusted residual = 3) significance level. A: Age; B: Lifestyle; C: Socio-economic status; D Health status

A

|  |  | Respondents’ Age | |  |
| --- | --- | --- | --- | --- |
| Criterion |  | $18-29$ | $30-59$ | $60 +$ |
| Mental handicap | Yes | 2.7 |  |  |
|  | NA |  |  | 2.5 |
| Psychological illness | Yes |  | $-2.5$ |  |
|  | No |  | 3.0 |  |
| Active in the community | Yes |  | $-2.9$ |  |
|  | No |  | 3.0 |  |
|  |  |  |  |  |
|  |  | Lifestyle | |  |
|  |  | Healthy | Average | Unhealthy |
| Mental handicap | Yes | 2.6 |  | $-2.7$ |
|  | No | $-2.3$ |  | 2.3 |

B

|  |  | Respondents’ Lifestyle | |  |
| --- | --- | --- | --- | --- |
|  |  | Healthy | Average | Unhealthy |
| Mental handicap | Yes | 2.6 |  | $-2.7$ |
|  | No | $-2.3$ |  | 2.3 |

C

|  |  | Respondents’ Socio-economic Status | | |
| --- | --- | --- | --- | --- |
|  |  | Lower | Middle | Higher |
| Life-threatening disease | NA |  |  | 3.5 |
| Senior citizens | Yes | 3.7 |  | $-3.3$ |
|  | No | $-3.9$ |  | 3.6 |
| Mental handicap | Yes | 2.2 |  | $-2.8$ |
|  | No | $-2.3$ |  | 2.5 |
| Psychological illness | Yes | 3.6 |  | $-3.2$ |
|  | No | $-3.6$ |  | 3.1 |
| Chronic illness | Yes | 2.4 |  | $-2.0$ |
|  | No | $-2.8$ |  |  |
| Working age | Yes | 2.0 |  |  |
|  | No | $-2.7$ |  |  |
|  | NA | 2.3 |  |  |
| Professional responsibility | No | $-2.7$ | 2.5 |  |
|  | NA | 3.1 | $-2.2$ |  |

D

|  |  | Health Status | | | |
| --- | --- | --- | --- | --- | --- |
|  |  | PCS $\geq54.2$ | PCS $<54.2$ | MCS $\geq57.3$ | MCS $<57.3$ |
| Life-threatening disease | Yes |  |  | 2.9 | $-2.9$ |
|  | No |  |  | $-3.1$ | 3.1 |
| Physical handicap | Yes |  |  | $-2.5$ | 2.5 |
|  | No |  |  | 3.1 | $-3.1$ |
| Low quality of life | Yes |  |  | $-2.7$ | 2.7 |
|  | No |  |  | 2.8 | $-2.8$ |
| Mental handicap | Yes |  |  | $-3.8$ | 3.8 |
|  | No | 2.7 | $-2.7$ | 3.6 | $-3.6$ |
| Social responsibility | Yes | $-3.3$ | 3.3 | $-3.6$ | 3.6 |
|  | No | 3.0 | $-3.0$ | 3.5 | $-3.5$ |
| Working age | Yes | $-2.8$ | 2.8 | $-3.2$ | 3.2 |
|  | No | 3.7 | $-3.7$ | 3.0 | $-3.0$ |
| Socially disadvantaged | Yes |  |  | $-2.8$ | 2.8 |
|  | No |  |  | 2.5 | $-2.5$ |
| Active in the community | Yes | $-2.5$ | 2.5 | $-2.5$ | 2.5 |
|  | No | 2.5 | $-2.5$ | 2.5 | $-2.5$ |
| Professional responsibility | Yes | $-3.0$ | 3.0 | $-4.0$ | 4.0 |
|  | No | 3.3 | $-3.3$ | 3.9 | $-3.9$ |
| Unemployed | Yes |  |  | $-3.1$ | 3.1 |
|  | No |  |  | 2.6 | $-2.6$ |

A positive value indicates that the respondents in this group gave significantly more often the respective response, while a negative value indicates that the respondents in this group gave that response significantly less often. NA stands for No Answer (Don’t know and Response refused combined). PCS and MCS in Table S3 D indicate the respondents’ physical and mental component summary, respectively.
