# Supplementary material for: Citizen Participation in Patient Prioritization Policy Decisions: An Empirical and Experimental Study on Patients' Characteristics
Source: PLoS One. 2012 May 9;7(5):e36824. doi: 10.1371/journal.pone.0036824 (PMC3348901; doi:10.1371/journal.pone.0036824)
Supplement: Table S3 — Logistic regression. (DOC) [file pone.0036824.s003.doc]

Table S3: Main effects for binary logistic regression (Chi Square values, p-values).

|  | Respondents’ characteristics | | | | |
| --- | --- | --- | --- | --- | --- |
| Criterion | Age | Socio-econ | Health Status | | Life Style |
|  |  |  | PCS | MCS |  |
|  | df = 1 | df = 2 | df = 1 | df = 1 | df = 2 |
| Life-threatening disease |  |  |  | 4.3, 0.039 |  |
| Children | 5.5, 0.019 | 7.4, 0.025 |  |  | 9.7, 0.008 |
| Senior citizens |  | 18.7, 0.000 |  |  | 6.7, 0.035 |
| Low quality of life |  |  |  | 4.5, 0.033 |  |
| With children | 4.3, 0.038 | 11.4, 0.003 |  |  | 8.4, 0.015 |
| Mental handicap | 11.7, 0.001 | 7.5, 0.024 | 7.3, 0.007 | 9.2, 0.002 | 17.9, 0.000 |
| Psychological illness |  | 17.1, 0.000 |  |  | 7.3, 0.027 |
| Social responsibilities |  |  |  | 6.4, 0.012 | 8.9, 0.012 |
| Socially disadvantaged |  |  |  | 4.1, 0.044 |  |
| Healthy lifestyle |  |  |  |  | 8.0, 0.018 |
| Professional responsibility |  |  |  | 7.8, 0.005 |  |
| Unemployed |  |  |  | 5.6, 0.018 |  |
