# Supplementary material for: Citizen Participation in Patient Prioritization Policy Decisions: An Empirical and Experimental Study on Patients' Characteristics
Source: PLoS One. 2012 May 9;7(5):e36824. doi: 10.1371/journal.pone.0036824 (PMC3348901; doi:10.1371/journal.pone.0036824)
Supplement: Table S4 — Odds ratio. (DOC) [file pone.0036824.s004.doc]

Table S4: Odds ratios and p-values for the covariates age, health status (PCS and MCS) and the factors socio-economic status and lifestyle. The factors levels for the socio-economic status were 1 for low, 2, for middle and 3 for high, the latter being the reference group. The factor levels for lifestyle were 1 for healthy, 2 for average and 3 for unhealthy, the latter being the reference group.

|  | Respondents’ characteristic | | | | |
| --- | --- | --- | --- | --- | --- |
| Criterion | Age | Health Status | | Socio-econ | Life Style |
|  |  | PCS | MCS |  |  |
| Life-threatening disease |  |  | 1.024, 0.032 |  |  |
| Children | 0.992,0.020 |  |  | G1: 1.549, 0.006  G2: 1.395, 0.036 | G =1, 1.585, 0.002 |
| Senior citizens |  |  |  | G1: 1.835, 0.000  G2: 1.413, 0.019 | G=1, 1.398, 0.001 |
| Low quality of life |  |  | 0.987, 0.034 |  |  |
| With children | 0.994, 0.038 |  |  | G1: 1.644, 0.001  G2: 1.429, 0.017 | G1: 1.426, 0.007  G2: 1.282, 0.022 |
| Mental handicap | 0.990, 0.001 | 0.985, 0.007 | 0.981, 0.003 | G1: 1.504, 0.007 | G1: 1.746, 0.000  G2: 1.331, 0.010 |
| Psychological illness |  |  |  | G1: 1.796, 0.000  G2: 1.381, 0.035 | G1: 1.352, 0.024  G2: 1.311, 0.015 |
| Social responsibilities |  |  | 0.984, 0.011 |  | G1: 1.434, 0.008 |
| Socially disadvantaged |  |  | 0.983,  0.040 |  |  |
| Healthy lifestyle |  |  |  |  | G1: 1.681, 0.032  G2: 1.736, 0.008 |
| Professional responsibility |  |  | 0.969, 0.004 |  |  |
| Unemployed |  |  | 0.971, 0.013 |  |  |
